# The BabySeq project: implementing genomic sequencing in newborns

**DOI:** 10.1186/s12887-018-1200-1

**Published:** 2018-07-09

**Authors:** Ingrid A. Holm, Pankaj B. Agrawal, Ozge Ceyhan-Birsoy, Kurt D. Christensen, Shawn Fayer, Leslie A. Frankel, Casie A. Genetti, Joel B. Krier, Rebecca C. LaMay, Harvey L. Levy, Amy L. McGuire, Richard B. Parad, Peter J. Park, Stacey Pereira, Heidi L. Rehm, Talia S. Schwartz, Susan E. Waisbren, Timothy W. Yu, The BabySeq Project Team, Pankaj B. Agrawal, Alan H. Beggs, Wendi N. Betting, Carrie L. Blout, Ozge Ceyhan-Birsoy, Kurt D. Christensen, Pamela Diamond, Dmitry Dukhovny, Kathryn E. Dunn, Shawn Fayer, Leslie A. Frankel, Casie A. Genetti, Chet Graham, Robert C. Green, Amanda M. Gutierrez, Maegan Harden, Margaret H. Helm, Lillian Hoffman-Andrews, Ingrid A. Holm, Joel B. Krier, Matthew S. Lebo, Kaitlyn B. Lee, Harvey L. Levy, Xingquan Lu, Sarah S. Kalia, Kalotina Machini, Amy L. McGuire, Jaclyn B. Murry, Medha Naik, Tiffany Nguyen, Richard B. Parad, Hayley A. Peoples, Stacey Pereira, Devan Petersen, Uma Ramamurthy, Vivek Ramanathan, Heidi L. Rehm, Amy Roberts, Jill O. Robinson, Serguei Roumiantsev, Talia S. Schwartz, Eleanor B. Steffens, Meghan C. Towne, Tina K. Truong, Grace E. VanNoy, Susan E. Waisbren, Caroline M. Weipert, Timothy W. Yu, Robert C. Green, Alan H. Beggs

**Affiliations:** 1 Division of Genetics and Genomics, The Manton Center for Orphan Disease Research, Boston Children’s Hospital, Boston, MA USA; 2 Department of Pediatrics, Harvard Medical School, Boston, MA USA; 3 Division of Newborn Medicine, Boston Children’s Hospital, Boston, MA USA; 4 Laboratory for Molecular Medicine, Partners Healthcare Personalized Medicine, Cambridge, MA USA; 5 Department of Pathology, Brigham and Women’s Hospital, Harvard Medical School, Boston, MA USA; 6 Department of Pathology, Memorial Sloan Kettering Cancer Center, New York, NY USA; 7 Division of Genetics, Department of Medicine, Brigham and Women’s Hospital, Boston, MA USA; 8 Harvard Medical School, Boston, MA USA; 9 Center for Medical Ethics and Health Policy, Baylor College of Medicine, Houston, TX USA; 10 Department of Psychological, Health and Learning Sciences, University of Houston College of Education, Houston, TX USA; 11 Department of Pediatric Newborn Medicine, Brigham and Women’s Hospital, Boston, MA USA; 12 Department of Biomedical Informatics, Harvard Medical School, Boston, MA USA; 13 The Broad Institute of MIT and Harvard, Cambridge, MA USA

**Keywords:** Newborn screening, Newborn sequencing, Whole exome sequencing, Methods, Randomized trial, Ethical, legal, social implications

## Abstract

**Background:**

The greatest opportunity for lifelong impact of genomic sequencing is during the newborn period. The “BabySeq Project” is a randomized trial that explores the medical, behavioral, and economic impacts of integrating genomic sequencing into the care of healthy and sick newborns.

**Methods:**

Families of newborns are enrolled from Boston Children’s Hospital and Brigham and Women’s Hospital nurseries, and half are randomized to receive genomic sequencing and a report that includes monogenic disease variants, recessive carrier variants for childhood onset or actionable disorders, and pharmacogenomic variants. All families participate in a disclosure session, which includes the return of results for those in the sequencing arm. Outcomes are collected through review of medical records and surveys of parents and health care providers and include the rationale for choice of genes and variants to report; what genomic data adds to the medical management of sick and healthy babies; and the medical, behavioral, and economic impacts of integrating genomic sequencing into the care of healthy and sick newborns.

**Discussion:**

The BabySeq Project will provide empirical data about the risks, benefits and costs of newborn genomic sequencing and will inform policy decisions related to universal genomic screening of newborns.

**Trial registration:**

The study is registered in ClinicalTrials.gov Identifier: NCT02422511. Registration date: 10 April 2015.

## Background

Clinical laboratories are increasingly offering genomic sequencing (next generation sequencing of the whole genome or exome), to diagnose rare disorders, individualize cancer treatments, and inform drug selection and dosing (pharmacogenomics) [1–10]. Moreover, experts anticipate that health systems will soon expand the use of genomic sequencing more broadly for disease risk assessment, carrier testing, prenatal screening, and potentially much more [11–15]. Genomic sequencing at a population level is rapidly becoming feasible and has the potential to revolutionize healthcare and improve patient outcomes.

Genomic sequencing may have its greatest lifelong impact on newborns. Not only can genomic sequencing facilitate diagnoses in sick newborns and infants, it has potential utility in newborn screening by identifying predispositions for future disease that can be mitigated through early intervention. In addition, data provided by genomic sequencing can be a resource for healthcare providers to query throughout an individual’s lifetime. The National Institutes of Health director Dr. Francis Collins has said: “…whether you like it or not, a complete sequencing of newborns is not far away,” [12] and the previous National Institutes of Child Health and Development (NICHD) director Dr. Alan Guttmacher explicitly invoked genomic sequencing of newborns: “One can imagine the day that 99% of newborns will have their genomes sequenced immediately at birth” [11]. As the President’s Council on Bioethics concluded as early as 2008, it may “…prove impossible to hinder the logic of genomic medicine from assimilating the currently limited practice of newborn screening into its all-embracing paradigm” [16].

This vision led the NICHD and National Human Genome Research Institute (NHGRI) to jointly issue a Request for Applications (RFA) to explore “opportunities to use genomic information for broadening our understanding of diseases identified in the newborn period.” Four groups were funded under this RFA and comprise the ***N****ewborn* ***S****equencing* ***I****n* ***G****enomic medicine and public* ***H****eal****T****h (NSIGHT)* consortium (https://www.genome.gov/27558493/newborn-sequencing-in-genomic-medicine-and-public-health-nsight/) [17]. The primary goal of our NSIGHT grant, the “**BabySeq Project”,** is to explore the medical, behavioral, and economic impacts of integrating genomic sequencing into the care of healthy and sick newborns. Here, we describe the design of the Project.

## Methods

### Study investigators

The BabySeq Project team includes a diverse group of investigators with expertise in genetics/genomics, neonatology, newborn screening, bioinformatics, molecular genetics, clinical trial design, ethics, and psychosocial, behavioral, and health outcomes measurement. The study includes an External Advisory Board with members drawn from clinical genetics, molecular genetics, neonatology, newborn screening, and ethics.

### Overview of study design

**Fig. 1:**
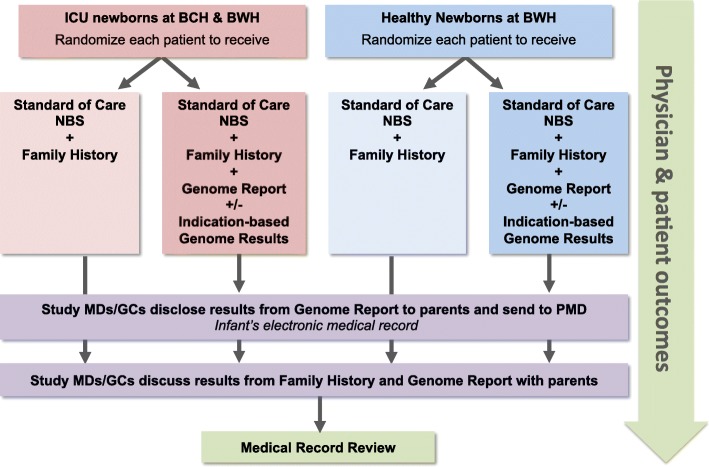
BabySeq Study Design Overview

The BabySeq Project study design was informed by a preexisting program, The MedSeq Project, [18–20] a randomized clinical trial assessing the impact of integrating genome sequencing into clinical medicine in adults. BabySeq is a randomized clinical trial that explores the impact of sequencing newborns in two cohorts, healthy and sick newborns (Fig. 1), and evaluates infant, family, and clinician outcomes. Within each cohort, families are randomized to a modified standard of care (family history and standard newborn screening [NBS]) or to a modified standard of care plus genomic sequencing. For those in the genomic sequencing arm a *Newborn Genomic Sequencing Report (NGSR)* is generated, which lists pathogenic or likely pathogenic variants in genes that have been strongly linked to childhood-onset diseases or diseases for which intervention is possible during childhood [21]. For newborns with a specific clinical presentation that potentially has a genetic etiology, a more in-depth analysis of the newborn’s sequence targeted to that presentation is available *(Indication Based Analysis, IBA)*. Parents complete surveys over the baby’s first year of life, and the baby’s provider/s complete surveys over the course of the study.

### The IRB and FDA

The BabySeq Project investigators are based at Boston Children’s Hospital (BCH), Brigham and Woman’s Hospital (BWH), and Baylor College of Medicine (BCM). All participant activities occur at BCH and BWH, and the IRBs at both institutions approved the protocol with the “greater than minimal risk with potential for benefit” risk determination. BCM collects and analyzes data on the ethics and psychosocial impact of newborn sequencing and was approved by their IRB through an expedited process.

The four NSIGHT studies underwent review by the Food and Drug Administration (FDA) and the procedure for sequencing, interpretation, reporting, and data collection in the BabySeq Project was determined to be a non-significant risk device study according to the investigational device exemptions (IDE) regulation (21 CFR 812).

### Rationale for a two cohort design to study sick and healthy newborns

A significant portion of newborns in the Neonatal Intensive Care Unit (NICU) have a condition with a genetic component [22]. Currently the most common practice for these cases is to send single or multiple gene tests until a diagnosis is made, potentially leading to delays in diagnosis and implementing appropriate care. Genomic sequencing immediately after birth may streamline the process of genetic testing by permitting the correct diagnosis to be made faster, potentially lowering hospitalization costs and improving clinical outcomes. Moreover, if additional symptoms develop, an already existing sequence can be re-interrogated and analysis targeted to those symptoms, leading to an answer more rapidly than ordering new genetic tests piecemeal. Although genomic sequencing is being increasingly used in sick children, including newborns, [23, 24] at many institutions the high cost and difficulty in obtaining reimbursement by insurance companies, [25] as well as uncertainty about the management of secondary findings, limits its use. As a result, studying the implementation of sequencing sick newborns remains a priority, as it is not currently accessible in many settings.

Sequencing healthy newborns may also provide parents with genetic information that predicts risk for genetic diseases. There is precedence for predictive genetic testing of newborns: state-mandated newborn screening identifies conditions for which early intervention improves outcomes [26]. Furthermore, predictive genetic testing is accepted in the care of children with a family history of a child-onset disorder, or disorders where there are preventative interventions available during childhood. The elective application of newborn genomic sequencing to healthy newborns expands on the newborn screening and predictive testing currently in place. In addition, identification of a newborn’s carrier status can facilitate parental testing and reproductive planning for the family.

### Rationale for a randomized design

A randomized controlled trial of whole exome sequencing (WES) vs. modified standard of care is an uncommon study design for genomic sequencing studies and provides a high degree of methodological rigor. This is important because concerns have been raised about the potential for negative psychosocial impact on families and health care providers of sequencing healthy newborns and returning results unrelated to a diagnosed medical condition, [27–29] and that unnecessary testing ordered by clinicians in response to the results could increase parental anxiety and health care costs [27]. Randomizing families allows us to evaluate the medical, economic, and behavioral outcomes related to parental impact and clinician decision-making in a manner while reducing biases generated by families that volunteer for the study.

### Population and recruitment

#### Population

The targeted enrollment for the BabySeq Project is approximately 200 newborns and their parents in each cohort: 1) healthy: the BWH Well Baby Nursery, and 2) sick: the BWH NICU, and BCH NICUs and other ICUs (see Table 1, inclusion and exclusion criteria). Within each cohort participants are randomized 1:1 WES:Standard of care. The newborn’s primary care provider and provider/s in the NICU/ICU are also invited to participate. For this sample size we estimate statistical power to be > 95% at α = 0.05 to test hypotheses that parents in the WES arm will report no greater personal distress or disruptions to parent-child relationships than parents in the control arm. We also estimate that we will have over 95% power to test hypotheses that parents in the WES arm will perceive greater utility in the information they receive than parents in the control arm.

**Table 1:** Inclusion and Exclusion Criteria

Inclusion criteria:	
Infants born at BWH and admitted to the Well Newborn Nursery, or to the BCH or BWH ICU	
At least one biological parent to have genetic counseling, donate DNA, and provide consent for testing the infant	
Exclusion criteria:	
Parents are non-English speaking	
Parents unwilling to have genomic reports placed in the medical record or sent to their primary care pediatrician	
Mother or father younger than 18 years of age	
Mother or father with impaired decisional capacity	
Age of infant is older than 42 days	
One of a multiple gestation	
Any infant in which clinical considerations preclude drawing 1.0 ml of blood	
Clinical exome ordered before the time of enrollment	
Missing consent of either biological parent (if known) or rearing parent (if applicable)	

#### Recruitment

##### Newborns and their parents

The BabySeq research staff first screen the newborns/families to determine eligibility. Permission to approach an eligible family is obtained from health care staff in the clinical unit. Parents are introduced to the study by the staff from the clinical unit and/or the BabySeq project. Interested families complete a pre-enrollment information session with a genetic counselor to learn about the study.

##### Health care providers

Parents provide the name of their newborn’s primary care provider. Health care provider/s of sick newborns include BWH and BCH Neonatology faculties, who were invited to enroll at the beginning of the study. Additional specialist care providers are identified by the parents, and by the research staff through the electronic medical record. All providers are contacted and asked to complete a baseline survey. Regardless of whether or not they complete a baseline survey, all primary care providers and providers involved in a newborn’s care during the course of the study are asked to complete an online post-disclosure survey.

It should be noted that participation of the newborn’s provider/s is optional and non-participation does not disqualify the newborn and family from enrollment or continuation in the study.

#### Consent

The consent process for the families starts with a pre-enrollment information session conducted by a genetic counselor, which includes an overview of study logistics, basic genetics education, review of types of reportable results, and a discussion of risks and benefits. After the session and prior to signing the consent form, the parents are administered 18 consent-understanding questions and incorrect responses are reviewed with the parents. Consent is required from both biological parents, if known, and from non-biological legal guardians, if applicable. Following consent, each parent receives a baseline survey. At least one parent must complete the baseline survey within 14 days in order to confirm study participation, providing families time to consider the study following discharge from the hospital. Families who do not complete a baseline survey are considered to have passively withdrawn from the study. Once one baseline survey is completed, the newborn is considered fully enrolled and is randomized to a study arm.

For providers, completing the survey constitutes consent to the study.

#### Parents who decline to participate in the BabySeq project

Parents who decline upon initial approach or after the pre-enrollment information session, are offered a brief “decliner survey” that queries their reasons for declining.

### Data and sample collection at enrollment

A detailed 3-generation pedigree is obtained from the parents. One mL of blood by venipuncture is collected from the newborn, divided into two 0.5 mL aliquots. Saliva samples are collected from both biological parents, unless not possible (e.g., anonymous sperm or egg donation).

### Review of medical records and family history report

Parents provide medical record releases for the newborn’s pediatric records, state newborn screening results, and the mother’s obstetric records. Records of subsequent care are requested when the infant is 6 weeks old and are reviewed in preparation for the results disclosure session. Medical records are requested and reviewed on an annual basis.

### Genomic sequencing

For newborns randomized to the genomic sequencing arm, DNA obtained from one of the 0.5 mL blood samples is used for WES; the second 0.5 mL aliquot is held as a back-up. An aliquot of the DNA is sent to the CLIA-certified Clinical Research Sequencing Platform at the Broad Institute, Cambridge, MA where WES is performed on an Illumina HiSeq platform. Variant interpretation and reporting is performed at the CLIA-certified Partners HealthCare Laboratory for Molecular Medicine (LMM), Cambridge, MA. Variants are filtered and classified according to previously described approaches [19] and professional guidelines [30]. Genes are classified using the Clinical Genome Resource (ClinGen) Gene Curation Working Group framework (https://www.clinicalgenome.org/curation-activities/gene-disease-validity/). Variants to be returned are confirmed by Sanger sequencing or digital droplet PCR. The average length of time from DNA extraction to completion of the report is 16 weeks.

If testing the parents could aid in the interpretation of a variant in the newborn, e.g., determining de novo occurrence, or determining the phase of two variants identified in a recessive gene, DNA is extracted from the parents’ saliva samples and Sanger sequencing of the variant is performed. Parental origin is not routinely determined for carrier variants found in the newborn. Parental DNA does not undergo WES.

### Reporting

A *Newborn Genomic Sequencing Report (NGSR)* is generated for newborns randomized to the genomic sequencing arm that includes pathogenic and likely pathogenic variants that indicate risk, or carrier status, for highly penetrant conditions presenting and/or managed during childhood. We anticipated that approximately 5% of newborns would have a reportable monogenic disease risk variant [31–33] and that roughly 90% will be a carrier for a reportable condition [19, 20]. Given the prevalence of carrier status, this allows us a greater opportunity to observe short-term reactions, health care expenditures, short-term medical benefits, potential effects on parental bonding, and how such information affects parents’ reproductive decisions. Additionally, pharmacogenomic variants in genes with strong evidence for relevance in medications used in the childhood period (e.g. *RYR1*, *G6PD*, or *TPMT* variants) are included on the NGSR.

An IBA is performed and included on the NGSR for sick newborns with a specific indication at the time of enrollment, or if a genetic indication is revealed through the record review or later in follow-up for any subject. This analysis, unlike the NGSR, also contains variants of uncertain significance (VUS) in genes associated with the indication.

Results are signed-out by American Board of Medical Genetics and Genomics (ABMGG)-certified clinical molecular geneticists. (See Ceyhan-Birsoy, et al., 2016 [21] for a description of gene curation).

The NGSR structure and content is based on the Genome Reports developed for the MedSeq project [19, 20]. The first page has a “*results summary*” of the findings followed by an “*interpretation summary*”, which includes “*monogenic disease risk variants*” and “*carrier status variants*” sections. The reported findings are summarized in a table that includes information on the disease, inheritance, gene transcript, variant, allele state, classification, and penetrance. If the parents were tested for a variant found in the newborn, the parent of origin is included in the table. If an IBA was requested, the “*interpretation summary*” includes “*variants relevant to the indication for testing*” and a similar table summarizing details, including coverage statistics for particular genes associated with that indication. Finally, there is a “*recommendations*” section. The next page of “*detailed variant information*” has additional detail about the variant, disease, familial risks, and reproductive risk. This organizational structure allows participants and providers easy access to the important information, and to the details if desired.

### Disclosure

Results for both arms of the study are disclosed to parents during an in-person session at the BWH or BCH by a study genetic counselor and physician. The family is told which arm they are in, and there is a discussion of the family history report (written by the genetic counselor based on the pedigree obtained at enrollment) and the standard NBS report. Parents in the sequencing arm also receive the NGSR, and results of an IBA (if performed). A study physician (most of whom are trained in clinical genetics) performs a physical examination to identify dysmorphic features or minor anomalies that might have been previously missed, and infants in the control arm who may have benefited from sequencing. Families are given a copy of the family history report, NBS report, and, for those in the sequencing arm, the NGSR.

### Reporting in the medical record and to providers

After disclosure of results, the genetic counselor and physician prepare a note summarizing the visit. This note, along with the family history report, NBS report, and, for those in the sequencing arm, the NGSR, are mailed to the parents and faxed to the infant’s pediatrician and other providers. These documents are uploaded to the infant’s medical record at BWH or BCH. Electronic reports are also available through a GeneInsight Clinic instance where physicians are notified of any variant classification changes [34–37].

### Outcomes

#### Outcomes addressed throughout the development and execution of the study

We have created a multi-step process in the clinical domain, where one has not existed before, providing comprehensive sequencing of newborns in a randomized controlled trial format. Development of this process, encompassing 1) protocol development, 2) recruitment and enrollment, 3) genomic sequencing, 4) analysis of the sequencing data in an organized and timely manner, 5) report generation, 6) return of the findings to participants and providers, and 7) placement of the information in the medical records. In addition we will assess economic outcomes, which is in and of itself, an important element of this study. The development and implementation of an effective workflow will provide important information on what works and what the pitfalls are.

Additional outcomes include:

- Socioeconomic and demographic characteristics of parents choosing to enroll in a newborn genomic sequencing study.
- The process and rationale for choice of genes and variants to report, which of those findings should be included or excluded, and categories of information (e.g., dominant adult onset conditions) that are not being returned but perhaps should be.
- Assessing optimal formats for reporting genomic results.
- Contributions of genomic data to medical management of infants in the ICU.
- Cost differentials of genomic sequencing between sick and healthy babies.
- Identification of hidden but discoverable phenotypes in babies that have risk variants, and if they are not immediately perceivable (as in infants with cardiac risks), the presence of “subclinical phenotypes” that can be explored.
- Medical, behavioral, and economic impacts of integrating genomic sequencing into the care of healthy and sick newborns.

#### Medical, behavioral and psychosocial outcomes

To objectively measure the impact of genomic newborn sequencing on parents and care providers, the goals are:

1. To compare the impact on parents of receiving a NGSR vs. standard of care, using clinical data and surveys measuring psychological and psychosocial impact, perceived utility, and behavioral responses.
2. To evaluate the experience and actions of the clinicians who receive the genomic reports compared to standard care.

BabySeq addresses these goals by analyzing clinical data and surveying parents and clinicians. Medical outcomes include time to final diagnosis, time to initiation of optimal therapy, length of hospital stay, and survival. Building on previous research [20], BabySeq also collects outcome data on key domains, including attitudes and preferences, healthcare utilization, health behaviors and intentions, decisional satisfaction, and psychological impact (Table 2). The project employs validated measures when possible, but the nature of the BabySeq Project and its study population required revised or novel measures for some outcomes where there were no existing instruments.

**Table 2:** Variable Domains

Variable Domains	Parents				Physicians		
	Baseline	Post-Disclosure	3-Mo	10-Mo	Baseline	Post-Disclosure	End of Study
Attitudes	X		X		X		X
Confidence	X	X		X	X		X
Perceived Utility	X		X	X	X	X	X
Genetic Perception	X			X	X		X
Sociodemographics	X				X		
Healthcare Utilization						X	
Report Utilization						X	
Information Seeking						X	
Preparedness & Interest					X	X	
Parent-Child Relationship	X	X	X	X			
Personal Distress (Depression/Anxiety)	X	X	X	X			
Child-Centered Stress			X	X			
Partner Relationship	X	X	X	X			
Perceptions of Child	X	X	X	X			
Health Behaviors & Intentions	X	X	X	X			
Social Support		X		X			
World View	X		X	X			
Health History	X						
Trust	X						
Satisfaction		X	X	X			
Understanding & Recall		X					

**Fig. 2:**
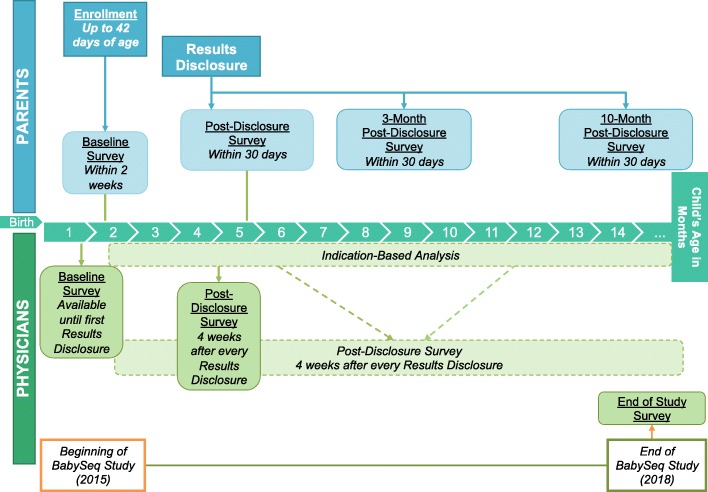
BabySeq Parental and Physician Survey Timeline

Parent surveys are administered at four time points over the infant’s first year of life: enrollment, following the results disclosure, and 3 and 10 months after results disclosure (Fig. 2). Because the BabySeq Project is specifically investigating the risks and benefits of genomic sequencing in the newborn period, the surveys address the psychosocial impact of sequencing on parent-child and parent-parent relationships during this critical formative period [38]. Family Systems Theory suggests that an event that affects one member of a family will affect the entire family system [39]. Therefore, the parent surveys assess parents’ perceptions of their child, child-centered stress, parent-child relationships, partner relationships, and parental depression and anxiety.

Provider surveys assess their knowledge, attitudes, and perspectives concerning genomic information at enrollment and at the study end. Each time they receive a NGSR, providers also complete a survey assessing their attitudes toward the results and their recommendations for follow-up healthcare.

Both parent and providers receive a monetary incentive for completing the surveys.

#### Economic outcomes

Economic outcomes associated with sequencing of newborns are collected. Medical record reviews and diagnoses are collected in all subjects over the first year of life. Cost data related to diagnostic laboratory testing and other medical procedures, medical visits, as well as parental time lost from work will be compared between the sequenced and control arms.

### Recording adverse events

Standardized questionnaires for depression or anxiety are included in each survey. If a parent receives a score above the cut-off for being clinically at-risk on instruments measuring depression or anxiety, or endorses the statement “The thought of harming myself has occurred to me/Thoughts that you would be better off dead or of hurting yourself in some way” (Edinburgh Postnatal Depression Scale//Patient Health Questionnaire – 9), they were contacted by the study psychologist (SW) to ensure that they had adequate supports and were not in danger of self-harm or hurting the baby. The study psychologist or genetic counselor may refer the parent to their primary care provider, a mental health professional, or emergency room if indicated or requested.

### Recruitment

Through an iterative process of periodic assessment, we have maximized enrollment. Initial enrollment predictions for the BabySeq Project were based on a hypothetical project similar to the BabySeq Project that our group previously reported [40] where nearly 85%.of parents approached in the BWH Well Baby Nursery were at least somewhat interested in the hypothetical possibility of their newborn undergoing WES. However, our actual enrollment rate has been significantly lower, leading us to identify and address hurdles to enrollment. Early on we discovered that one of the primary logistical hurdles was the short time frame for enrollment, since healthy newborns are generally discharged by 48 h of life, which does not give parents who are busy caring for their newborn much time to consider their decision to enroll and complete the baseline survey. We adapted by providing parents 2 weeks after discharge to complete the baseline survey, allowing them time to consider their decision and complete the survey outside of the hectic post-partum environment.

Additionally we instituted a “decliner survey” and based on assessment of the results changed some procedures to be less burdensome, including allowing parents to return for a consent session after discharge, and changing the 10-month post disclosure in-person visit to a survey and phone check-in with a genetic counselor.

### Consent process

There was concern that some parents might not understand the potential implications for their family of having their newborn sequenced. To address this issue, we instituted the brief post-counseling survey to ensure parents understood the core information from the counseling session.

### Criteria for reporting

In advance of enrollment, we reviewed over 1400 genes for strength of disease association, inheritance pattern, age of onset, and penetrance, with approximately 800 meeting criteria for return in the Project [17]. As variants in un-curated genes are identified in participants, they need to be assessed in real time. In addition, because new information regarding a gene’s role in disease is constantly reported in the literature, we update the curation as each new potentially pathogenic variant is identified. As a result, some variants in genes initially not on the returnable list may be reclassified as returnable, while others may be removed. While the reference gene list is not utilized for variant filtration, the pre-curated data significantly reduces the time spent on results interpretation, since it allows the assessment process for pre-curated genes to focus solely on reviewing any new information that became available since the last update [17].

### Assessing outcomes

As we decided on outcome measures we needed to strike a balance between obtaining a comprehensive picture of parents’ experiences and minimizing the burden with long surveys and multiple questionnaires. In addition, because newborns ages 0–6 weeks of age are enrolled, the study subjects vary in age at each survey time point, creating complications in designing age-appropriate measures for each encounter. In addition, newborns randomized to the genomic sequencing arm will have results of varying nature and severity and we needed to be thoughtful in how we compare the experiences of families who received results with differing degrees of impact. Ideally, we will be following these families longitudinally beyond the first year of life, since we recognize that collecting data for only the first year of life is a short time period.

## Discussion

The BabySeq project is a study of the implementation of WES in newborns, and uses a randomization scheme that will allow us to definitively address some of the concerns that have been raised in this field about potentially negative psychological impacts of presenting genomic information to families of newborn infants. The study is complex and the process of designing and beginning implementation will lead to several important insights into the best ways to deliver genomic medicine in a newborn setting.

As genomic sequencing becomes further integrated into clinical care, the incorporation of genomic sequencing into universal newborn screening becomes a real possibility. The BabySeq Project will provide objective data regarding the risks and benefits of newborn genomic sequencing in terms of the health impacts on the child, the psychosocial implications for the family, and the ways in which clinicians use the information. This study also provides preliminary information about the economic impact and burden on the healthcare system of newborn genomic sequencing. We hope that the results from the BabySeq Project will inform policy decisions related to universal genomic screening of newborns.

## Abbreviations

ABMGG: American Board of Medical Genetics and Genomics
BCH: Boston Children’s Hospital
BCM: Baylor College of Medicine
BWH: Brigham and Woman’s Hospital
CLIA: Clinical Laboratory Improvement Amendments
ClinGen: Clinical Genome Resource
DNA: Deoxyribonucleic acid
FDA: Food and Drug Administration
IBA: Indication Based Analysis
IDE: Investigational device exemptions
IRB: Institutional Review Board
LMM: Laboratory for Molecular Medicine
NBS: Newborn Screening
NGSR: Newborn Genomic Sequencing Report
NHGRI: National Human Genome Research Institute
NICHD: National Institutes of Child Health and Development
NICU: Neonatal Intensive Care Unit
NSIGHT: Newborn Sequencing In Genomic Medicine and Public Health
PCR: Polymerase chain reaction
RFA: Request for Applications
VUS: Variants of Uncertain Significance
WES: Whole Exome Sequencing
